# Phosphorus—A Peculiar Symptom

**Published:** 1898-06

**Authors:** J. B. Campbell

**Affiliations:** Brooklyn, N. Y.


### PHOSPHORUS—A PECULIAR SYMPTOM.

J. B. Campbell, M. D., Brooklyn, N. Y.

In September, 1896, was called to Mr. L., a hard drinker, aged 48 years, who had just had two epileptic seizures about fifteen minutes apart. I arrived in time to see him in a third attack. He had all the usual appearances. The aura seemed to be accompanied by a pain in the chest, becoming more severe as the attack approached. There was much darting of the tongue and licking of the lips.

He would answer questions during this stage, but after a while his glance became vacant and then the convulsions suddenly seized him. The face was dark purple, eyes turned violently upward, chewing of the tongue; tonic, changing to clonic spasms.

The most peculiar feature of the case was the termination of the convulsive attack. The muscular motions seemed to centre in the hips, which were jerked violently up and down, having exactly the same appearance as at the completion of the sexual orgasm.

Having in mind the symptoms of a case described by Dr.

F. H. Lutze in a paper read before the Brooklyn Hahnemannian Union, in which Phos. had relieved similar jerkings, I rapidly reviewed the features of the case and gave that remedy. There were no more convulsions, the patient recovered, and has had no return.

Acon., and some other remedy which I do not now remember, had been given between the first few attacks; but the Phos. was followed by a return to consciousness and a disappearance of all convulsive and other symptomatic manifestations.

